# The Impact of Obesity on Colorectal Surgery: A Survey of Canadian Surgeons

**DOI:** 10.4021/gr370w

**Published:** 2011-09-20

**Authors:** Nader Azer, Richdeep S. Gill, Xinzhe Shi, Chris deGgara, Daniel W. Birch, Shahzeer Karmali

**Affiliations:** a Department of Surgery, University of Alberta, Edmonton, Alberta, Canada; b Center for the Advancement of Minimally Invasive Surgery (CAMIS), Royal Alexandra Hospital, Edmonton, Alberta, Canada; c These authors contributed equally to this work

**Keywords:** Colorectal cancer, Canadian surgeons, Obesity, Colon resection

## Abstract

**Background:**

Over 1.7 billion adults worldwide are considered overweight or obese, with the prevalence of obesity in Canada increasing rapidly. Obesity has been shown to affect surgical outcomes such as local recurrence of cancer and wound infections following colorectal surgery. The objective of this study was to determine the perception/attitudes of Canadian surgeons toward the impact of obesity on the practice of colorectal surgery.

**Methods:**

A twenty-question survey was administered to Canadian surgeons through mail and email solicited via the Canadian Association of General Surgeons over a period of 2010-2011. The questions focused on surgeon demographics, experience with laparoscopic colon resections and their perception of the impact of obesity toward surgical proficiency and complications.

**Results:**

One hundred seventy-seven Canadian surgeons completed the survey. There was a wide range of experience among surgeons in terms of years of practice and number of colon resections performed per year. The majority (72.9%) reported having primary general surgical training. A majority of surgeons (57.7%) identified obesity as a risk factor for colorectal surgery. Furthermore, a majority agreed that obesity is a risk factor for wound infection (97.2%), stomal retraction (90.4%) and stomal herniation (82.5%). While obesity was not considered a contraindication to laparoscopic colon surgery, it was considered to increase operative time (98.3%), cardiovascular (80.2%) and respiratory (95.4%) complications.

**Conclusion:**

The majority of surgeons across Canada believe obesity is a risk factor for post-operative complications following laparoscopic colorectal surgery. However, the majority did not consider obesity a contraindication for laparoscopic colon resection. Surgical and peri-operative colorectal protocols may need to be re-assessed to identify methods to manage the obese patient more effectively.

## Introduction

The prevalence of overweight (BMI ≥ 25 kg/m^2^) and obese individuals (BMI ≥ 30 kg/m^2^) has increased from 15% to 35% over the last three decades [1]. With an estimated 1.7 billion individuals classified as either overweight or obese, the management of obesity is a rapidly growing concern [2]. Respectively, in Canada, over 60% of adults have been classified as overweight or obese [3]. This change in the weight and adiposity of the patient population has affected many different areas of medicine. The impact of obesity is now an important consideration in surgical patients.

According to the World Health Organization, colorectal cancer remains the third most common cancer in the world in both men and women [4]. Recent evidence has highlighted an association between obesity and colorectal cancer [5]. Obesity has been correlated with both surgical and oncologic outcomes [6]. Furthermore, increased postoperative complications related to obesity have been reported [7]. Nevertheless, limited literature exists to correlate how Canadian surgeons are applying evidence-based medicine linking obesity to colorectal cancer and colorectal cancer surgery. Our objective was to assess the perspective of Canadian surgeons on the impact of obesity in Canada on surgical outcomes and complications of colorectal cancer.

## Subjects and Methods

An anonymous online questionnaire was made available to all active surgeons who are members of the Canadian Association of General Surgeons (CAGS). Surgeons received reminder emails through the monthly CAGS e-news for four months asking them to complete the survey. Specifically the “monthly CAGS e-news” consisted of an email send out by the association to all members of CAGS and the link for the survey website along with the brief explanation of the survey was embedded in text of the monthly news. To increase number of respondents the survey was sent via mail on two separate occasions. The first mailing of the survey corresponded with membership renewal forms, in attempt to increase viewership.

**Figure 1:**
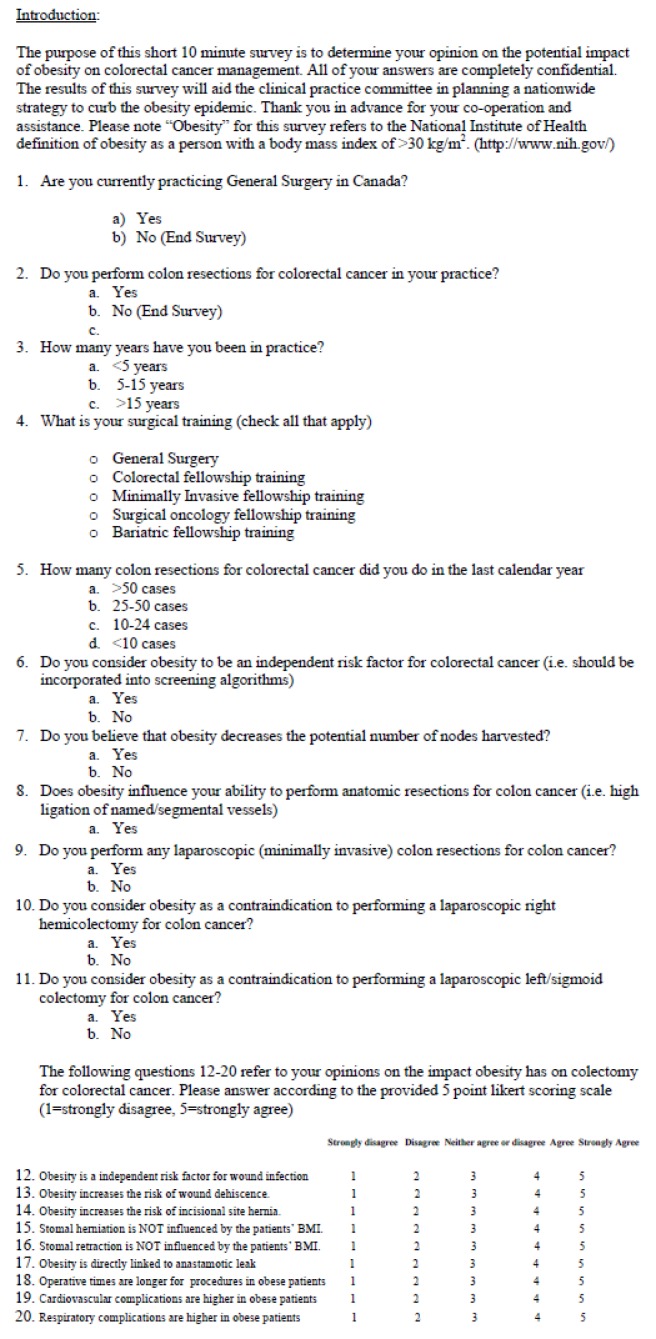
Questionnaire.

The questionnaire was designed with input from members of the CAGS clinical practice committee (CG and SK) and the CAGS bariatric working group (DWB and SK). Evidence to design the study came from recent literature evaluating obesity and colorectal surgery [5-8]. It consisted of twenty questions following a brief introduction, as shown in Figure 1. Exclusion criteria from the questionnaire were based on a “No” response to either the first or second question. In another words, the surgeon needed to be a currently practicing in Canada, and performing colorectal resections. At the conclusion of the four-month period all answers were tabulated and results expressed as percentages.

## Results

A total of 203 Canadian surgeons responded online or via mail to our questionnaire from estimated 1200 CAGS active members, which gives a response rate of approximately 17%. Of the total 203 responses, 26 met our exclusion criteria (surgeons not currently practicing in Canada or not performing colorectal resections), thus 177 responses were included for analysis.

**Figure 2:**
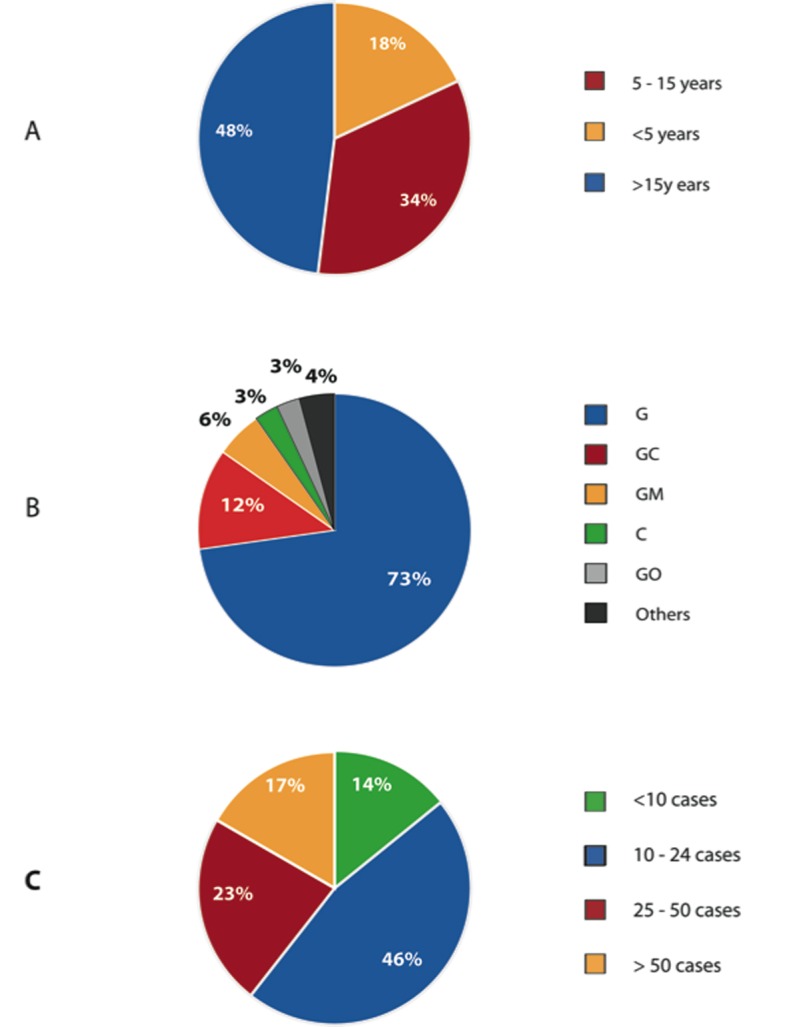
Demographics of Canadian Surgeons. (A) Number of years in practice. (B) Level of training of Canadian Surgeons. (C) Number of colorectal resections performed last year in their practice.

**Figure 3:**
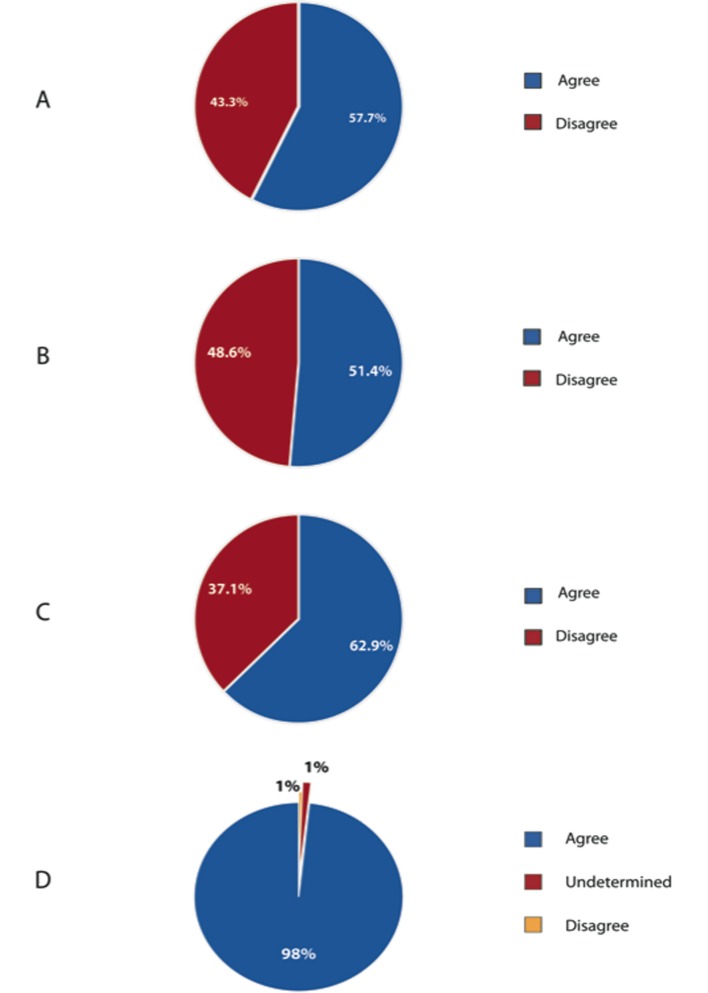
Perception of the effect of obesity and colorectal cancer and colorectal resection. (A) Obesity as a risk factor for colorectal cancer. (B) Effect of obesity on number of lymph nodes harvested. (C) Effect of obesity on performing anatomical resection. (D) Effect of obesity on length of procedure.

As seen in Figure 2, responding Canadian surgeons had a range of clinical surgical experience, with 73% performing colorectal resections following completion of a general surgery residency. 46% of Canadian surgeon respondents performed 10 to 24 resections per year, with another 40% performing more than 25 colorectal resections per year. 57.7% of Canadian surgeon respondents agreed that obesity is an independent risk factor for colorectal cancer (Fig. 3). A large majority, 98% agreed that obesity lengthens operative time for colorectal resection.

**Figure 4:**
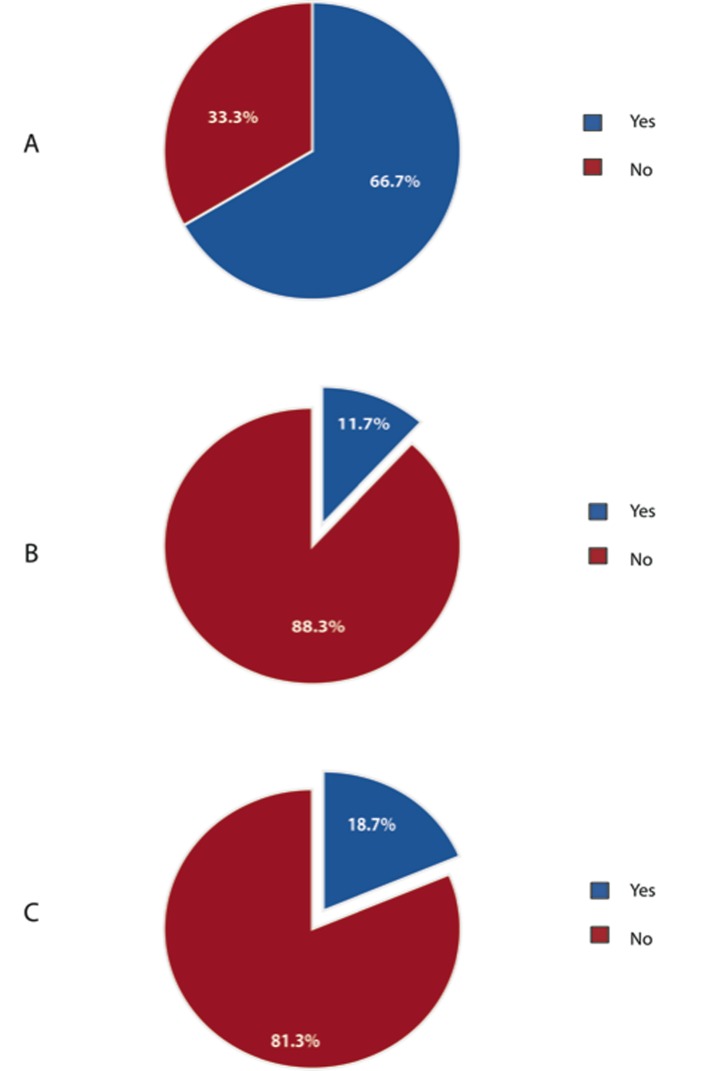
Laparoscopic colorectal resections. (A) Percentage of Canadian surgeons performing laparoscopic colorectal resections. (B) Obesity as a contraindication for laparoscopic right hemicolectomy for cancer. (C) Obesity as a contraindication for laparoscopic left hemicolectomy for cancer.

As shown in Figure 4, 66.7% of respondents were performing laparoscopic colorectal resection as part of their surgical practice. Furthermore, 88.3% and 81.3% of respondents did not consider obesity to be a contraindication to laparoscopic right and left hemicolectomy, respectively.

**Figure 5:**
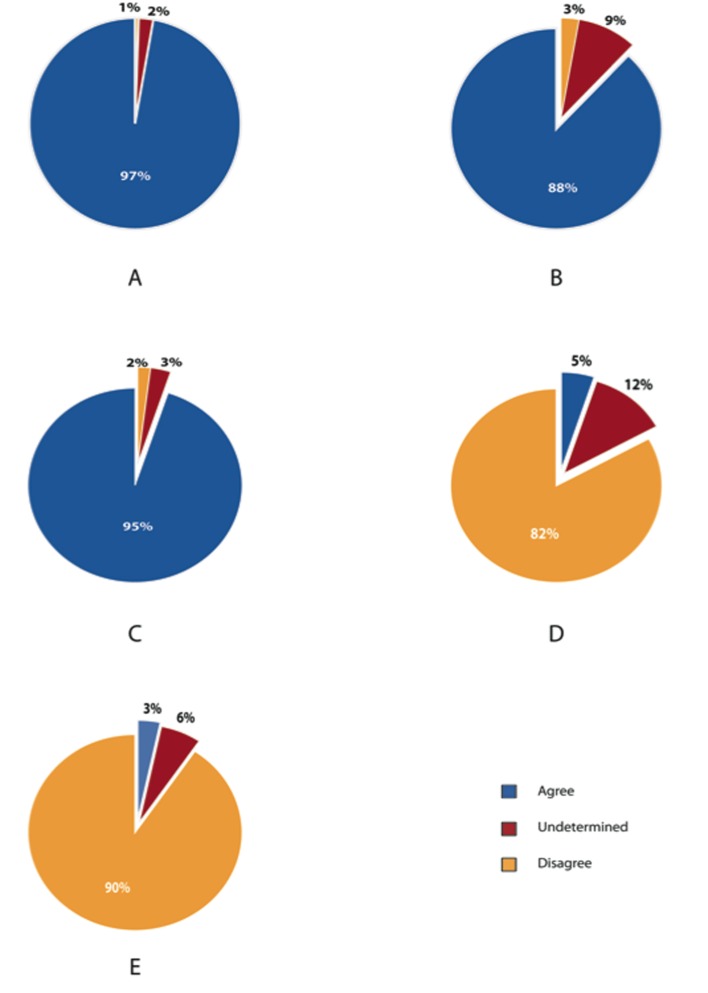
Postoperative wound and stomal complications related to obesity. (A) Obesity as an independent risk factor for wound infection. Obesity increases risk for (B) wound dehiscence (C) incision site hernia. (D) Stomal herniation is not influenced by patients BMI. (E) Stomal retraction is not influenced by patients BMI.

Ninety seven percent of respondents agreed that obesity is an independent risk factor for postoperative wound infection (Fig. 5). Of Canadian surgeon respondents, 88% and 95% identified obesity as a risk factor for wound dehiscence and incision site herniation, respectively. 82% and 90% of respondents disagreed that stomal herniation and stomal retraction are not influenced by the patient’s BMI.

**Figure 6:**
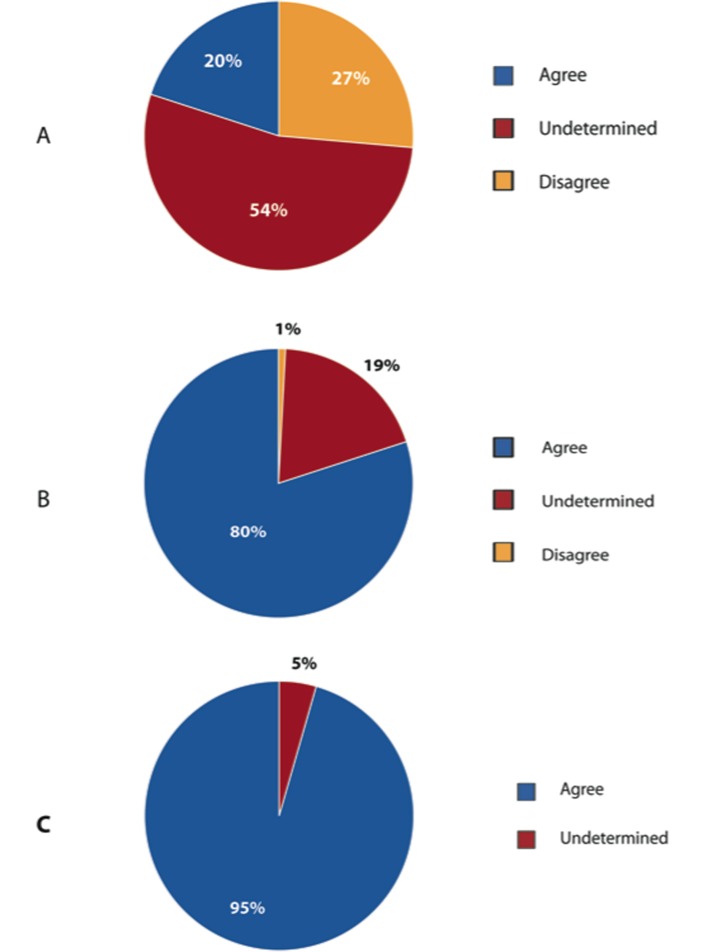
Major postoperative complications related to obesity. (A) Obesity directly linked to anastomotic leak. (B) Cardiovascular complications are higher in obese patients. (C) Respiratory complications are higher in obese patients.

As seen in Figure 6, a majority of respondents (54%) remained uncertain about whether obesity is directly linked to anastomotic leakage. 80% and 95% of respondents agreed that cardiac and respiratory complications are more likely in obese patients.

There was no significant difference of results in all questions regarding years of practicing, fellowship training, volume of colorectal surgery.

## Discussion

According to this study, there appears to be a diverse range of Canadian surgeons performing colorectal resections for colorectal cancer. A slight majority of Canadian surgeon respondents perceived obesity as an independent risk factor for colorectal cancer and an influence on anatomical colorectal resection. According to our questionnaire, a majority of surgeons identified obesity as risk factor for major cardiopulmonary and postoperative wound complications. However, the majority of surgeon respondents did not perceive obesity as a contraindication for laparoscopic colon resection.

It is interesting that Canadian surgeon respondents identified obesity as an independent risk factor for colorectal cancer approximately 55% of the time. A large cohort study of 145 000 patients in Austria suggested that obesity may be associated with an increased risk of colon cancer in men [9]. They reported an increased hazard ratio (HR) of 1.56 for male obese class I patients (BMI > 30 kg/m^2^) and a HR of 2.48 for male obese class II patients (BMI > 35 kg/m^2^). A larger prospective study including 368 277 men and women from nine European countries, also identified BMI > 29.4 kg/m^2^ as increasing the risk of developing colorectal cancer in males by 1.55 times compared to BMI < 23.6 kg/m^2^ [10]. However, the same increase in relative risk was not seen in women with the same BMI. Interestingly, waist circumference greater than 103 cm was associated with an increased risk of colorectal cancer in both men and women. A meta-analysis by Dai et al, reported a pooled relative risk (RR) of 1.37 (95% CI: 1.21 - 1.56) for colorectal cancer in overweight and obese men [5]. Similar to other studies, overweight and obese women were not identified to have an increased RR for colorectal cancer. Unfortunately, none of the included studies in this meta-analysis incorporated population data from Canada. Furthermore, as epidemiologic studies, these studies cannot comment on the mechanism underlying this association. Thus, the perception of Canadian surgeons may be accurate, that based on current literature, it has not been clearly determined if the Canadian obese population is at increased risk. Also, in our questionnaire, we did not specify the sex to which the question was referring, which may have led to some ambiguity.

In a large majority of surgeon respondents, obesity was identified as risk factor for postoperative wound complications. A retrospective comparison of obese (BMI > 27 kg/m^2^) and non-obese patients undergoing colorectal resection between 1990-1997, reported significantly greater wound infections (13% versus 4%, P < 0.05) in obese patients following rectal resection [11]. However, this study included colorectal resections for a variety of different pathologies. Balentine et al recently retrospectively reviewed 150 cases of rectal cancer resection, which included 42 minimally invasive (MIS) rectal resections [7]. They reported increasing superficial wound infections with increasing BMI in both the open and MIS rectal surgery. This group also reported increased operative time in the MIS surgery group with increasing BMI, suggesting a correlation. Interestingly, close to 99% of Canadian surgeon respondents perceived increased operative times with obesity. In another retrospective review of 162 laparoscopic colorectal resections, wound infections were more frequent in obese (12.9%) compared to non-obese patients (3.1%) [12]. Furthermore, this study reported greater number of major complications in obese patients (52%) versus non-obese patients (12%). Major complications were defined as complications leading to prolonged hospital stay (i.e. myocardial infarction and pneumonia). Tsujinaka et al also reported increased wound infection in obese patients following laparoscopic sigmoid colectomy for colon cancer [13]. However, they quantified visceral fat area as their definition of obesity. Operative time was also shown to be significantly greater by approximately 30 min in obese patients compared to non-obese patients.

The majority of Canadian surgeon respondents related obesity to stomal complications. A prospective analysis of 97 patients for stoma complications identified high BMI based on univariate regression to be associated with more stomal retractions and early skin excoriation [14]. Duchesne et al also identified obesity as a risk factor for stomal complications based on multivariate analysis of 164 patient records [15]. Though there is a lack of strong evidence, it seems Canadian surgeons are likely aware of the increased potential for stomal complications in obese patients.

A majority of Canadian surgeon respondents also identified obesity as a risk factor for wound dehiscence. According to an American College of Surgeons dataset from 121 hospitals in the US, morbid obesity (BMI > 35 kg/m^2^) was shown to increase the odds of wound dehiscence by 3.5 times compared to normal weight patients (BMI < 24 kg/m^2^) following colon resection for cancer [16]. However, obesity (BMI > 30 kg/m^2^) did not significantly increase the odds of wound dehiscence. Furthermore, this study also reported increased odds of wound infection and pulmonary embolism following colon resection for cancer in morbidly obese patients. However, myocardial infarction was not identified as a more frequent complication. Benoist et al reported increased cardiac complications following right hemicolectomy in obese patients, but this difference was not observed after left hemicolectomy [11]. This highlights the lack of evidence available to draw definitive conclusions regarding the association of obesity and postoperative cardiopulmonary complications following colorectal surgery for cancer.

Despite the strong perceived association between obesity and postoperative complications including wound infections, wound dehiscence, stomal complications and cardiopulmonary morbidity, the majority of Canadian surgeon respondents did not identify obesity as a contraindication for laparoscopic colon resection. Interestingly, a minority identified obesity as a contraindication; unfortunately our questionnaire does not allow further exploration of the specific reasons.

### Limitations

All surveys such as ours are limited by response rate, response bias, and question ambiguity. In terms of our response rate, an estimated 1200 active CAGS members potentially received our questionnaire. Of this, we had approximately 17% respond either online or by mail. In most epidemiologic surveys, a response rate of 30% is considered reasonable. Though our response rate is lower than our initial goal of 35%, it may be possible that it represents a greater proportion than expected. It is unknown the number of Canadian surgeons (CAGS members) that are performing colorectal resection in obese patients. However, it is known that the CAGS membership represents a large diverse group of general surgeons, with various subspecializations. Thus, of the 1200 CAGS members, it is unlikely that all perform colorectal resection for colon cancer. It is also likely that a limited number of surgeons not performing colorectal resections would respond to our questionnaire. Therefore, our response rate may be higher that the calculated 17%, but this cannot be definitely proven. However, with minimal Canadian literature in this area, we believe it is important to gauge the general perception of obesity and colorectal cancer in Canada. By emailing the CAGS members along with the monthly CAGS e-news over a four-month period we attempted to maximize the viewership our survey. Furthermore, we mailed the questionnaire in the same envelope as membership renewal information and forms to increase the chances of survey completion.

Secondly, it is difficult to remove response bias from a survey to assess the perception of certain populations, in our case Canadian surgeons. It typically is more likely for surgeons to respond to a questionnaire that they have a strong interest in. Thus, our results may represent the perceptions of surgeons with stronger opinions, either positive or negative. Fortunately, most of our questions produced either split responses (almost 50% on either side), or large majorities (80-90%). Thus, the results we have reported may accurately represent the general perception of Canadian surgeons.

Thirdly, ambiguity of survey questions is difficult to completely remove. Our goal was to simplify and clarify the included questions, to minimize misinterpretations. Both the CAGS clinical practice committee (CG and SK), the CAGS bariatric working group (DWB and SI), designed the questionnaire and pre-tested it on small number of surgeons in an attempt to remove vague questions. Furthermore, both committees are well versed with recent literature in this area to create well-informed and reasonable questions.

### Conclusion

Based on our questionnaire, Canadian surgeons appear to be uncertain regarding the association of obesity and colorectal cancer risk. However, the majority of surgeons across Canada identify obesity as a risk factor for post-operative complications following colorectal resection. Nonetheless, the majority did not consider obesity a contraindication for laparoscopic colon resection.
